# Correlation between fat-soluble vitamin levels and inflammatory factors in paediatric community-acquired pneumonia: A prospective study

**DOI:** 10.1515/med-2024-0972

**Published:** 2024-06-07

**Authors:** Jianyuan Liao, Lifang Zhang, Gangxin Chen, Yuxing Luo

**Affiliations:** Department of Blood Transfusion, Zhongshan Hospital of Xiamen University, School of Medicine, Xiamen University, Xiamen, China; Assisted Reproduction Laboratory, Fujian Provincial Maternity and Children Hospital, Affiliated to Fujian Medical University, Fu Zhou, China; Department of Joint Surgery and Sports Medicine, Zhongshan Hospital of Xiamen University, School of Medicine, Xiamen University, Xiamen, China; Emergency Department Fujian Provincial Maternity and Children Hospital, Affiliated to Fujian Medical University, Fu Zhou, China

**Keywords:** paediatric CAP, fat-soluble vitamins, TNF-a, IL-1, IL-10

## Abstract

Community-acquired pneumonia (CAP) is a common respiratory disease in children. This prospective cohort study of 110 children with CAP and 100 healthy children investigated the relationship between the levels of vitamin A, D and E and inflammatory markers, such as tumour necrosis factor (TNF-a), interleukin-1 (IL-1), interleukin-10 (IL-10), neutrophils (NE) and C-reactive protein (CRP), in CAP. The haemoglobin, leukocyte concentration, NE, monocytes and CRP concentration in the CAP group showed significant differences (*P* < 0.05). The levels of vitamin A, D and E in the CAP group were lower than those in the control group, while the levels of TNF-a and IL-1 were higher than in the control group; the differences were statistically significant (*P* < 0.05). The IL-10 levels showed no significant differences (*P* > 0.05). Pearson analysis revealed that the vitamin A, D and E levels were all correlated with the TNF-a, IL-10 and CRP levels (*P* < 0.05). The vitamin A, D and E levels of the CAP children were lower than those of the healthy children. Thus, the content of fat-soluble vitamins is correlated with the secretion of TNF-a and IL-10. The research provides a new direction for the prevention, diagnosis and treatment of CAP.

## 1 Introduction

Community-acquired pneumonia (CAP) in children is a prevalent respiratory disease primarily caused by mixed infections of various pathogens [1]. CAP remains a leading cause of early childhood mortality in many regions due to children’s susceptibility and low immunisation rates [2,3]. Currently, CAP diagnosis relies on clinical symptoms, chest radiography and pathogen detection in respiratory secretions [4]. Additional diagnostic aids beyond standard methods are needed. Some studies suggest a correlation between vitamin levels and the treatment and prevention of CAP [5–7], while others argue that vitamin supplementation alone may not reduce pneumonia risk [8]. The fat-soluble vitamins are vitamins A, D, E and K [9]. Vitamins A, D and E are closely related to obesity and inflammation in adolescents [10].

As a class of small molecule peptides, cytokines perform various biological activities, playing a key role in anti-infection, anti-tumour and apoptosis induction, as well as participating in the related diseases of immune inflammation under specific conditions [11]. Cytokines are essentially proteins or glycoprotein hormones, and they can be divided into either type I or type II according to different cytokine receptors: type I cytokines include colony-stimulating factor and interleukin; type II cytokines include tumour necrosis factor (TNF)-α/β and interleukin (IL)-10 [12]. Inflammatory mediators like cytokines TNF-a, IL-1, IL-10 and C-reactive protein (CRP) play key roles [13,14].

This study focuses on analysing the levels of the three most common lipid-soluble vitamins in CAP patients aged 4–9 years and explores their relationship with inflammatory mediators in paediatric CAP.

## 2 Methods

### 2.1 Sample size

According to relevant reports, the incidence of childhood CAP is as high as 30% [15]. Previous experiences in our hospital have shown that 20% of paediatric CAP patients have insufficient vitamin levels. The sample estimation software PASS11 was used to calculate the ideal sample size with a two-sided *t* test (*α* = 0.05, *p* = 0.2, *β* = 0.2), and the result was 62 subjects. If the rate of loss for the follow-up was 20%, the sample was 62/0.8 = 76 subjects. We set the same number for the control group and the experimental group (*n*1 = *n*2 = 76).

### 2.2 Data

**Figure 1:**
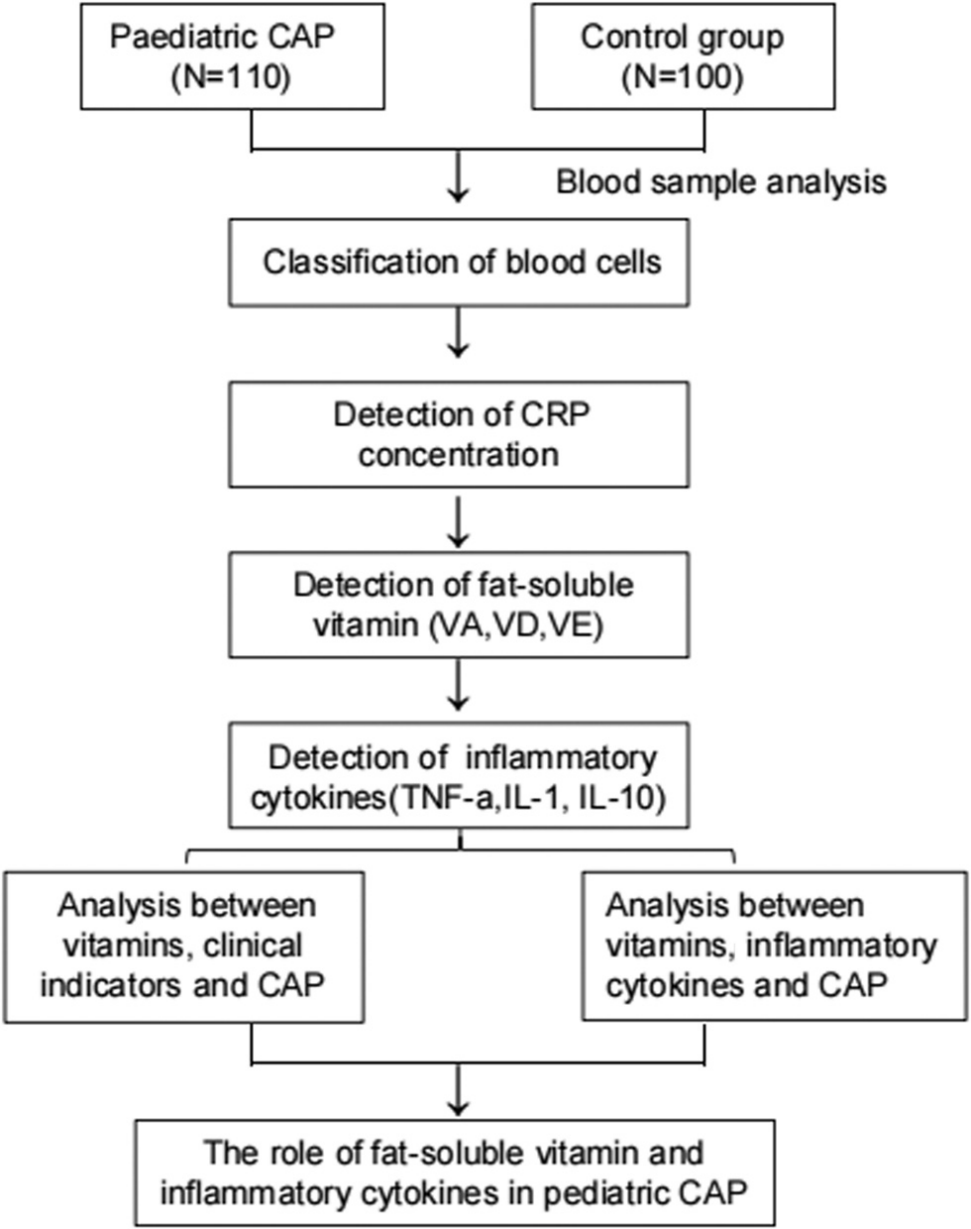
Flow chart: study design.

A total of 350 paediatric cough patients who visited the emergency department between January 2019 and January 2021 were initially included in the study. After excluding patients with respiratory diseases other than CAP and those who had previously undergone treatment or declined to participate, a final cohort of 110 patients diagnosed with CAP was studied. The recruitment of patients took place in a specialised children’s hospital emergency room located in an urban area with high rates of childhood vaccination. Additionally, 100 healthy children aged 4–9 years who had undergone routine physical examinations at our hospital were randomly selected as negative controls. To minimise individual variations, children within the age range of 4–9 years were chosen, while factors such as sex, height and weight were standardised (Figure 1).

### 2.3 CAP diagnostic and exclusion criteria

When diagnosing CAP in children, it is important to consider the following criteria: recent cough, sputum production, worsening respiratory symptoms with purulent sputum, fever (axillary temperature >37.5℃), presence of wet rales in the lungs, elevated leukocyte or neutrophil count and radiographic evidence of dense or interstitial changes in the chest. In this study, all patients exhibited a combination of symptoms from criteria one to four along with the fifth criterion [16,17].

Patients with hospital-acquired pneumonia, pulmonary tuberculosis, lung tumour, pharyngitis, bronchitis and other upper respiratory tract diseases were excluded. In addition, patients who had recently taken various forms of vitamins or folic acid supplements were excluded. Furthermore, individuals with lesions in adjacent organs of the lung, abnormal immune function or those with a long history of chronic disease were also excluded.

### 2.4 Detection of blood cells

Peripheral blood samples (3 mL) from the CAP patient and control groups were collected in EDTA anticoagulant tubes for leukocyte classification and other cell analysis (RBC, Hb) using the XN-3000 analyser (Sysmex, Germany).

### 2.5 Detection of CRP concentration

Whole blood samples (20 μL) were analysed for CRP concentration using a BC5390 Proteometer (Mindray Biomedical Electronics Co., Ltd, Shenzhen, China).

### 2.6 Detection of fat-soluble vitamin levels

Plasma was isolated by high-speed centrifugation, and serum samples (200 μL) were analysed for vitamin A, D and E concentrations using HPLC (Agilent, USA).

### 2.7 Detection of related inflammatory cytokines

Serum concentrations of cytokines TNF-a, IL-1 and IL-10 were measured using an ELISA kit in a microplate reader (Edkang Biotech Co., Ltd, Yantai, China) following the manufacturer’s instructions (Enzyplate Biotechnology Co., Ltd, Jiangsu, China).

### 2.8 Statistical analysis

Data analysis was performed using SPSS 17.0 statistical software (IBM, Armonk, NY, USA), with the measurement data in mean ± standard deviation. Cases and controls were analysed using an independent sample *t*-test or chi-square test, and correlation analysis was performed using Pearson analysis. For all tests, a significance level of *P* < 0.05 was employed, and any result with a *P*-value lower than 0.05 was considered statistically significant.


**Ethical approval:** The ethics committee of our hospital reviewed and approved the study (ethics committee registration number: 2019KY029). All methods were performed in accordance with the relevant guidelines and regulations.

## 3 Results

### 3.1 Clinical data between the patients and the control group

No significant differences were observed in the prevalence of anaemia between the two groups (RBC >4 × 10^12^/L). In addition, there were no statistical variances in age, sex ratio, body mass index (BMI), first diagnosis rate or medication history between the patients and the control group (*P* > 0.05). Haemoglobin (Hb) levels were lower in the patient group compared to the control group, while white blood cell (WBC), neutrophils (NE) percentage, monocyte (MO) percentage and CRP levels were higher, with statistically significant differences (*P* < 0.05; Table 1).

**Table 1:** Comparison of the fundamentals between the two groups (*n* = 210)

	CAP group *n* = 110	Control group *n* = 100	*t*/*X*_2_	*P*
Age (years)	5.78 ± 1.23	5.70 ± 1.18	0.48	0.62^a^
BMI	14.47 ± 3.07	14.46 ± 2.78	0.01	0.98^a^
Sex ratio (%)	59.10 (65/45)	53.00 (53/47)	0.79	0.37^b^
First diagnosis rate (%)	95.50 (105/5)	98.00(98/2)	−1.05	0.30^b^
RBC (10^12^/L)	4.98 ± 0.28	5.01 ± 0.35	−0.56	0.48^a^
Hb (g/L)	135.21 ± 10.81	156.54 ± 13.68	−4.56	0.00^a^
WBC (10^9^/L)	11.52 ± 3.35	7.26 ± 2.16	31.25	0.00^a^
NE (%)	70.35 ± 10.54	51.21 ± 11.16	43.56	0.00^a^
LY (%)	20.35 ± 8.65	20.89 ± 10.35	0.45	0.71^a^
MO (%)	9.05 ± 1.65	6.04 ± 0.35	17.88	0.00^a^
BA (%)	1.03 ± 0.35	1.39 ± 0.48	0.75	0.39^a^
CRP (μg/mL)	14.71 ± 10.43	0.39 ± 0.22	55.28	0.00^a^

Notes: ^a^Independent samples *t*-test; ^b^Chi-squared test; *P* < 0.05 indicates statistical significance. Abbreviation: SD, standard deviation. BMI, body mass index; RBC, red blood cells; Hb, haemoglobin; WBC, white blood cell; NE, neutrophils; LY, lymphocytes; MO, monocyte; BA, basophils; CRP, C-reactive protein.

### 3.2 Comparison of fat-soluble vitamins and inflammatory factors between patients and the control group

A comparison of fat-soluble vitamins and inflammatory factors between the patients and control group revealed that the levels of vitamins A, D and E were lower in the CAP group compared to the control group. Furthermore, the levels of TNF-a and IL-1 were found to be significantly higher in the CAP group than in the control group (*P* < 0.05). The level of cytokine IL-10 in the CAP group was marginally higher compared to the control group; however, this difference did not reach statistical significance (*P* > 0.05; Table 2).

**Table 2:** Comparison between fat-soluble vitamins and inflammatory factors in the two groups (*n* = 210)

	CAP group *n* = 110	Control group *n* = 100	*t*	*P*
VA (μg/mL)	0.41 ± 0.19	0.48 ± 0.18	−10.13	0.00
VD (ng/mL)	87.55 ± 8.71	89.89 ± 11.38	−6.49	0.00
VE (μg/mL)	17.92 ± 7.79	20.68 ± 10.02	−8.63	0.00
TNF-a (pg/mL)	46.38 ± 9.73	30.06 ± 6.13	56.84	0.00
IL-1 (pg/mL)	8.86 ± 2.53	6.03 ± 1.48	38.71	0.00
IL-10 (pg/mL)	34.27 ± 7.60	33.87 ± 7.21	1.51	0.13

Notes: Independent samples *t*-test; *P* < 0.05 indicates statistical significance.

### 3.3 Analysis of the correlation between vitamins and inflammatory factors in paediatric patients with CAP

**Figure 2:**
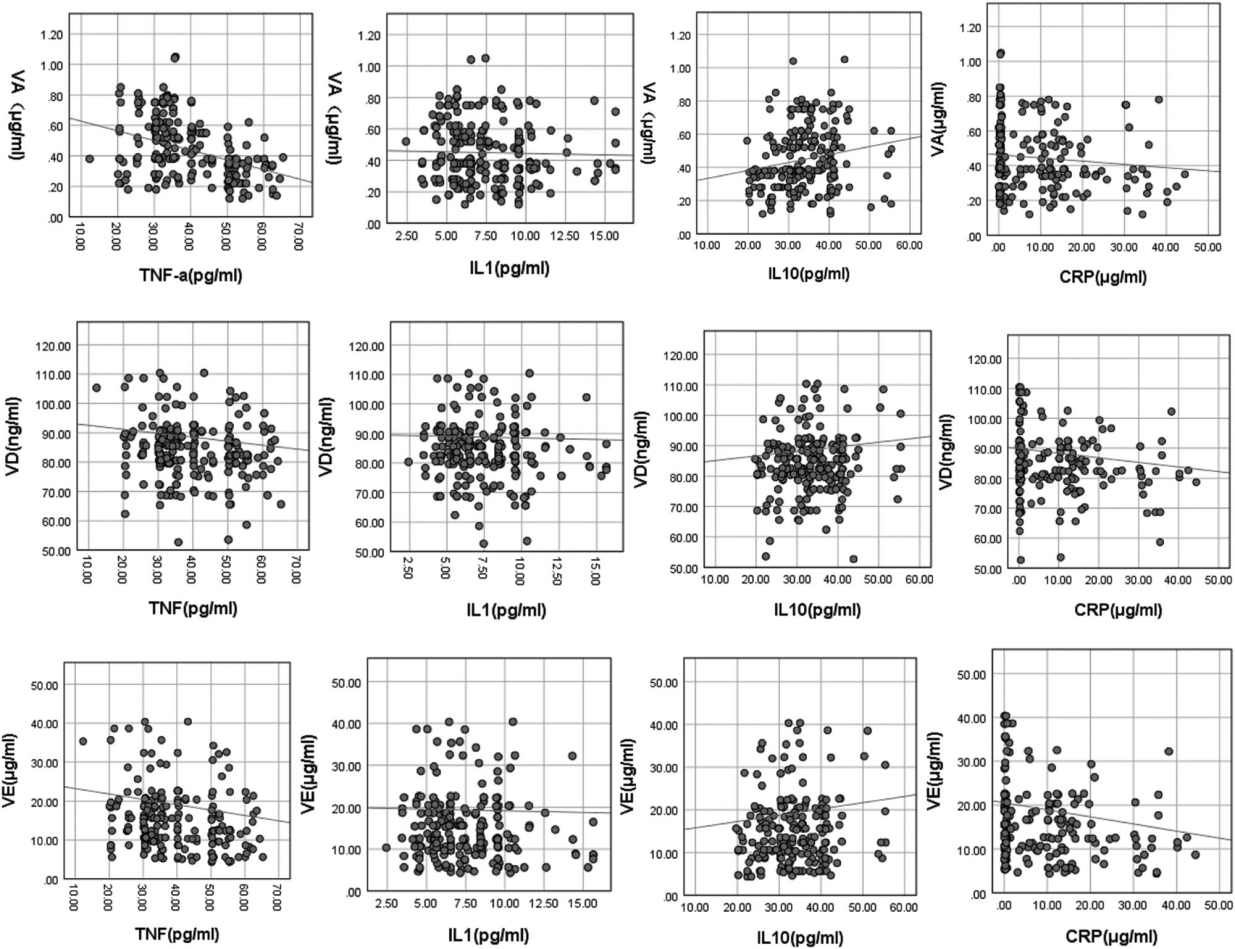
Correlation between fat-soluble vitamins and inflammatory factors in paediatric CAP patients.

Paediatric CAP exhibited a notable correlation between vitamin A and IL-1 and TNF-α (*P* < 0.05), a positive correlation between vitamin D and IL-10 and CRP (*P* < 0.05) and a positive correlation between vitamin E and IL-10 and CRP (*P* < 0.05; Table 3 and Figure 2).

**Table 3:** Analysis of the correlation between vitamins and inflammatory factors in pediatric CAP patients (*n* = 210)

	TNF-a (pg/mL)		IL-1 (pg/mL)		IL-10 (pg/mL)		CRP (μg/mL)	
	*r*	*p*	*r*	*p*	*r*	*p*	*r*	*p*
VA (μg/mL)	−0.378	0.000	−0.024	0.167	0.183	0.000	−0.098	0.000
VD (ng/mL)	−0.151	0.000	−0.027	0.134	0.110	0.000	−0.154	0.000
VE (μg/mL)	−0.175	0.001	−0.022	0.225	0.120	0.000	−0.181	0.000

Notes: Pearson test; *P* < 0.05 indicates statistical significance. A negative correlation between vitamin A, D, E and TNF-a, CRP in pediatric CAP patients and a positive correlation between IL-10 (*P* < 0.05).

### 3.4 Correlation analysis between fat-soluble vitamins and cytokines in children

There was no significant correlation between the absorption of vitamin A and the absorption of vitamin D and E in children (*P* > 0.05); however, there was a significant correlation between the absorption of vitamin D and vitamin E (*P* < 0.05). Furthermore, there was a notable correlation among various cytokines (*P* < 0.05; Tables 4 and 5).

**Table 4:** Analysis of the correlation between vitamins (*n* = 210)

	VA (μg/mL)		VD (ng/mL)		VE (μg/mL)	
	*r*	*p*	*r*	*p*	*r*	*p*
VA (μg/mL)	1.000	—	0.041	0.107	0.037	0.144
VD (ng/mL)	0.041	0.107	1.000	—	0.958	0.000
VE (μg/mL)	0.037	0.144	0.958	0.000	1.000	—

Notes: Pearson test; *P* < 0.05 indicates statistical significance. There was a significant correlation between the absorption of vitamin D and vitamin E (*P* < 0.05).

**Table 5:** Analysis of the correlation between cytokines (*n* = 210)

	TNF-a (pg/mL)		IL-1 (pg/mL)		IL-10 (pg/mL)		CRP (μg/mL)		
	*r*	*p*	*r*	*p*	*r*	*p*	*r*		*p*
TNF-a (pg/mL)	1.000	—	0.017	0.504	−0.129	0.000	0.058	0.021	
IL-1 (pg/mL)	0.017	0.504	1.000	—	−0.093	0.000	0.109	0.000	
IL-10 (pg/mL)	−0.129	0.000	−0.093	0.000	1.000	—	-0.002	0.924	
CRP (μg/mL)	0.058	0.021	0.109	0.000	−0.002	0.924	1.000	—	

Notes: Pearson test; *P* < 0.05 indicates statistical significance. There was a significant correlation between TNF-a and IL-10, CRP. There was a significant correlation between IL-1 and IL-10, CRP (*P* < 0.05).

## 4 Discussion

CAP is a common infectious disease in children, presenting with symptoms such as fever, cough, infection and dyspnoea [15]. Viral, mycoplasma and bacterial infections, as well as decreased immunity due to malnutrition, are believed to be the primary causes of CAP in children [18,19]. Some studies have demonstrated that fat-soluble vitamin deficiency in children is correlated with respiratory and digestive system diseases, infectious diseases and allergic diseases [20]. Vitamins have been shown to influence the expression of inflammatory and anti-inflammatory factors, thereby bolstering immune function. Furthermore, vitamins can enhance the differentiation of immune cells and promote the phagocytosis of bacteria upon respiratory pathogen invasion [9,10]. Some studies have indicated a potential link between vitamin deficiencies and the development of CAP [7,21].

Vitamins are categorised as either lipid-soluble or water-soluble. Some research studies have established the standard levels of fat-soluble vitamins in children, with recommended levels for vitamins A (>0.30 μg/mL), D (20–100 ng/mL) and E (>7 μg/mL) [22–24]. Our study found that although vitamin levels in both the CAP patients and the control children met the criteria described above, vitamin A, D and E levels in the paediatric CAP patients were significantly lower than those in the controls (*P* < 0.05). This disparity suggests a general deficiency of vitamins in CAP patients within this region. Interestingly, a correlation was observed between vitamin D and E absorption in children aged 4–9 years; however, this correlation was not seen with vitamin A. This finding aligns with previous reports, although the underlying reasons remain unspecified in the literature [25].

Of the 110 patients included in this study, 93 exhibited inflammatory manifestations, representing 84.5% of the population, and all patients experienced a febrile response. The auxiliary examinations predominantly indicated elevated leukocyte counts, increased percentage of NE and heightened CRP levels. Inflammatory response serves as the primary clinical feature in CAP patients, acting as the body’s defence mechanism against bacterial, viral and other foreign invaders. Inflammation onset relies heavily on immune cell activation and the release of inflammatory factors, such as TNF-a, IL-1, IL-6, IL-8 and IL-10. TNF-a and IL-1 are categorised as proinflammatory factors, while IL-10 is classified as an inflammatory suppressor [13,14]. The study findings revealed higher levels of TNF-a and IL-1 in paediatric CAP patients compared to healthy individuals, with no significant difference in IL-10 levels. This disparity may be attributed to the fact that these patients were all newly diagnosed, leading to immune activation upon initial virus invasion, resulting in lower IL-10 levels, indicative of immunosuppression. These results suggest that one can monitor the therapeutic effect of CAP patients through the detection of inflammatory factors. Pearson analysis indicated a correlation between fat-soluble vitamin levels (A, D and E) and cytokine levels (TNF-a and IL-10) in CAP patients, suggesting a close relationship between vitamin levels and immunomodulatory regulation.

The presence of certain vitamin levels in the body is essential for maintaining metabolism. Despite their small quantity in the body, vitamins play a crucial role in maintaining immune self-stability, immune surveillance, immune defence and other functions [26]. In recent years, there has been increased awareness of the importance of purposeful vitamin supplementation in children’s diets. However, due to children’s higher demand for vitamins, lower absorption levels compared to adults, lack of self-discipline, picky eating habits and other factors, it is common to observe low vitamin levels in preschool children [22]. Insufficient levels of fat-soluble vitamins in children can have the following effects: (1) An impaired immune system response to infectious invasion and immune system dysfunction. Vitamins A, D and E and their analogues can be used to enhance therapeutic efficacy in immunocompromised children [27]. (2) Developmental disorders [22]. (3) Insufficiency of the gonads, thus resulting in a lack of hormone levels needed for body development [28]. (4) Childhood obesity leads to childhood vitamin absorption difficulties, which can then lead to body dysfunction [10].

The diagnosis and treatment of CAP in children present certain challenges, with inflammation playing a key role. Currently, oral antibiotics are the primary treatment for CAP, with severe cases requiring intravenous antibiotics [29]. Amoxicillin is commonly used as the first-line antibiotic in preschool children [29]. Research suggests that vitamins can enhance immune cell function by promoting the secretion of cytokines, which play a crucial role in inflammation. Adequate intake and supplementation of vitamins are important for both the prevention and treatment of CAP in children. Existing literature indicates that factors such as patient age, geographical location, nutritional status, socioeconomic conditions and immune function also significantly influence the occurrence of CAP in children, in addition to antibiotic use and vaccination [30].

The results of this study provide new insights for preventing CAP in children. However, due to the small sample size and various factors influencing vitamin absorption, this study has limitations. CAP occurrence is linked to patient immunity levels, environmental factors and the type of pathogens involved. Thus, the study’s sources of bias contributed to uncertainty in the results.

In conclusion, the results of this study indicate a correlation between the levels of vitamins A, D and E in children with CAP and the cytokines TNF-α and IL-10.
